# Biotinylated Bilirubin Nanoparticles as a Tumor Microenvironment‐Responsive Drug Delivery System for Targeted Cancer Therapy

**DOI:** 10.1002/advs.201800017

**Published:** 2018-04-24

**Authors:** Yonghyun Lee, Soyoung Lee, Sangyong Jon

**Affiliations:** ^1^ KAIST Institute for the BioCentury Department of Biological Sciences Korea Advanced Institute of Science and Technology (KAIST) 291 Daehak‐ro Daejeon 34141 Republic of Korea

**Keywords:** bilirubin nanoparticles, biotin transporters, reactive oxygen species (ROS), stimuli responsiveness, targeted cancer therapy, tumor microenvironments

## Abstract

The tumor microenvironment (TME) plays a crucial role in tumorigenesis and cancer cell metastasis. Accordingly, a drug‐delivery system (DDS) that is capable of targeting tumor and releasing drugs in response to TME‐associated stimuli should lead to potent antitumor efficacy. Here, a cancer targeting, reactive oxygen species (ROS)‐responsive drug delivery vehicle as an example of a TME‐targeting DDS is reported. Tumor targeting is achieved using biotin as a ligand for “biotin transporter”–overexpressing malignant tumors, and bilirubin‐based nanoparticles (BRNPs) are used as a drug‐delivery carrier that enables ROS‐responsive drug release. Doxorubicin‐loaded, biotinylated BRNPs (Dox@bt‐BRNPs) with size of ≈100 nm are prepared by a one‐step self‐assembly process. Dox@bt‐BRNPs exhibit accelerated Dox‐release behavior in response to ROS and show specific binding as well as anticancer activity against biotin transporter–overexpressing HeLa cells in vitro. bt‐BRNPs labeled with cypate, near‐infrared dye, show much greater accumulation at tumor sites in HeLa tumor‐bearing mice than BRNPs lacking the biotin ligand. Finally, intravenous injection of Dox@bt‐BRNPs into HeLa tumor‐bearing mice results in greater antitumor efficacy compared with free Dox, bt‐BRNPs only, and Dox@BRNPs without causing any appreciable body weight loss. Collectively, these findings suggest that bt‐BRNPs hold potential as a new TME‐responsive DDS for effectively treating various tumors.

The tumor microenvironment (TME), which acts as the “soil for seed”—in this case cancer cells—plays a critical role in promoting tumorigenesis, drug resistance, immunosuppression, and metastasis of solid tumors.^1^ The TME accumulates features that creates a unique physicochemical environment that is far different from that of normal tissue.[[qv: 1a,b,d,e]] Accordingly, a drug delivery system (DDS) capable of exploiting unique TME‐associated features, such as mildly acidic pH, elevated oxidative stress, and overexpression of certain enzymes, would be a logical strategy for effective cancer therapy.^2^ In keeping with this idea, numerous attempts have been made to develop a DDS that is responsive to the TME‐specific pH (≈6–6.5).^3^ This pH trigger has been shown to lead to accelerated drug release and greater antitumor efficacy compared with DDS lacking such a TME‐responsive drug‐release feature.^3^ In most tumors, cancer cells and various TME‐associated cells, including tumor‐associated macrophages, inflammatory cells, and myeloid‐derived suppressor cells, overproduce reactive oxygen species (ROS), thereby generating elevated oxidative stress in the TME.[[qv: 1b,c,4]] This elevated ROS in the TME can also be exploited by a DDS to release encapsulated drugs in response to ROS, facilitating the internalization of these agents in cancer cells and leading to higher antitumor efficacy.[[qv: 1a,b,e,5]] In fact, there have been a number of reports of DDS that exploit ROS‐responsive drug‐release characteristics.^6^ However, most such systems consists of de novo‐synthesized artificial materials that show slow responses to secreted ROS, thereby limiting their translation into the clinic and highlighting the need for the development of a fast ROS‐responsive DDS based on biocompatible and biodegradable naturally occurring materials.[[qv: 6a,b]] Furthermore, the ideal drug‐delivery candidate would not only possess ROS‐responsive drug‐release behavior, it should also exert an intrinsic anticancer effect to maximize therapeutic efficacy.

Bilirubin (BR), an endogenous bile pigment and the final metabolite in the heme catabolic pathway, acts as a potent antioxidant and immune modulator.^7^ Very recently, we provided the first report of bilirubin‐based nanoparticles (BRNPs), formed by the self‐assembly of PEGylated BR, and demonstrated that they are highly potent against various inflammatory diseases in vivo, suggesting their potential as a nanomedicine platform.^8^ Furthermore, we have shown that BRNPs undergo dual‐stimuli (light and ROS)‐responsive particle disruption, and thus have the potential to be used as stimuli‐responsive drug‐delivery carriers.^9^ In fact, doxorubicin (Dox)‐loaded BRNPs (Dox@BRNPs), when combined with near‐infrared (NIR) laser irradiation at 650 nm, show high antitumor efficacy in human lung carcinoma‐bearing mice, reflecting rapid drug release within the tumor induced by light‐triggered particle disruption.^9^ Interestingly, BRNPs alone show some level of antitumor efficacy owing to the intrinsic anticancer effect of BR on cancer cells.^9^ In our previous reports, we exploited the so‐called enhanced permeability and retention (EPR) effect to deliver drug‐loaded BRNPs into tumors and used external photoirradiation to induce rapid drug release to kill cancer cells.^9^ However, an NIR laser due to its tissue penetration limit is not a proper option to treat most solid tumors, which form deep inside the body, and the EPR effect alone is not sufficient to achieve specific, effective tumor targeting. Here we report cancer‐targeting ligand‐conjugated BRNPs encapsulating an anticancer drug as a ROS‐responsive, TME‐targeting DDS for cancer treatment. Among the numerous cancer‐targeting ligands, we chose the vitamin biotin (vitamin B_7_ or H) as a model ligand, given its previously demonstrated potential to target and bind “biotin transporter”–overexpressing tumors,^10^ such as rapidly proliferating malignant cancers; in addition, biotin is a simple, small molecule that is easy to modify.^11^ Our findings highlight the potential of biotinylated BRNPs encapsulating Dox (Dox@bt‐BRNPs) as a new DDS that targets the elevated oxidative stress in the TME of biotin transporter–positive tumors, and show that this system may lead to enhanced antitumor potency and efficacy by rapidly releasing drugs that penetrate and diffuse deep into the tumor and ultimately into cancer cells.

To prepare bt‐BRNPs, we synthesized two forms of PEGylated bilirubin: bt‐PEG_3400_‐BR and PEG_2000_‐BR (**Figure** **1**a; Figure S1, Supporting Information). A series of bt‐BRNPs with differing densities of the cancer‐targeting ligand was prepared by self‐assembly of bt‐PEG_3400_‐BR and PEG_2000_‐BR at different formulation ratios using the film‐formation and rehydration method (Figure S2a, Supporting Information). For the preparation of Dox@bt‐BRNPs, phosphate‐buffered saline (PBS) containing Dox was used during the rehydration step.^9^ Because the length of hydrophilic PEG in bt‐PEG_3400_‐BR is much longer than that in PEG_2000_‐BR, it is expected that upon self‐assembly of the two PEGylated BRs, the biotin‐bearing PEG can protrude out in the resulting PEGylated BRNPs, which may be able to minimize steric hindrance and interference of the low‐hanging PEG_2000_ layer in the biotin‐mediated cancer cell binding.^12^ As shown in Figure 1c, bt‐BRNPs (95 mol% PEG_2000_‐BR and 5 mol% bt‐PEG_3400_‐BR) were spherical‐shaped nanoparticles with a diameter of ≈80 nm. Dynamic light scattering measurements of bt‐BRNPs revealed a hydrodynamic size of ≈101 ± 9 nm and zeta potential of −18 ± 3 mV at concentrations greater than ≈10 × 10^−9^ m (Figure S2b,c, Supporting Information).

**Figure 1:**
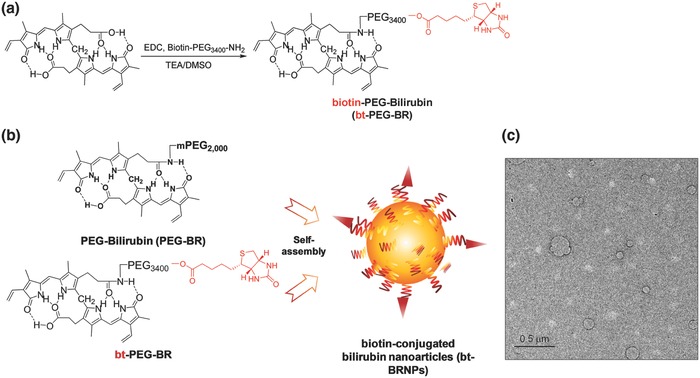
Biotin‐conjugated bilirubin nanoparticles (bt‐BRNPs) are formed from biotin‐PEG‐bilirubin (bt‐PEG‐BR) and PEG‐bilirubin (PEG‐BR). a,b) Scheme for the synthesis of bt‐PEG‐BR starting from free bilirubin and biotin‐PEG (a) and the formation of bt‐BRNPs by self‐assembly from PEG‐BRs and bt‐PEG‐BR in PBS (b). c) Transmission electron microscopy (TEM) images of bt‐BRNPs. Scale bar: 500 nm.

To determine the optimal density or ratio of bt‐PEG_3400_‐BR component in bt‐BRNPs for maximizing cancer cell targeting, we prepared a series of Dox‐loaded bt‐BRNPs (Dox@bt‐BRNPs) with different ligand density by using 1‐, 2‐, 5‐, and 10 wt% of bt‐PEG_3400_‐BR in the formulations. HeLa human cervical cancer cells and A549 human lung carcinoma cells, chosen as biotin transporter‐positive cancer cell lines,^11^ were treated with Dox@bt‐BRNPs or Dox@BRNPs for 1 h. As shown in fluorescent images of Dox, which possesses intrinsic fluorescence (Figure S3, Supporting Information), both 5 and 10 wt% biotinylated nanoparticles showed greater uptake of Dox by the two cancer cell types than other nanoparticle formulations. Because 5 wt% Dox@bt‐BRNPs was sufficient for the ligand optimization, we selected the formulation for use in subsequent in vitro and in vivo studies. To further confirm the targeting specificity of Dox@bt‐BRNPs, we treated a biotin transporter–negative fibroblast cell (NIH3T3) and the two biotin transporter–positive cancer cell lines, HeLa and A549, with Dox@BRNPs and Dox@bt‐BRNPs. As expected, the intensity of the Dox fluorescent signal of Dox@bt‐BRNPs was strong in both HeLa and A549 cells, whereas little signal was observed in biotin transporter–negative NIH3T3 cells (**Figure** **2**). Following treatment with non‐biotinylated Dox@BRNPs, Dox uptake in all three cell lines was similarly minimal. Furthermore, treatment of cells with an excess of free biotin (2 × 10^−3^ m) before Dox@bt‐BRNPs treatment dramatically reduced drug uptake, even by biotin transporter–positive cancer cells, suggesting competition for binding to the biotin transporter between free biotin and bt‐BRNPs. Despite numerous examples of biotin‐mediated drug delivery for cancer therapy, the mechanism of how biotin‐tagged therapeutics or nanoparticles can be taken up by cancer cells is still poorly understood. A piece of evidence found in the previous reports,^13^ however, suggests that after binding of the biotin‐tagged nanoparticles or therapeutics to the biotin transporter (i.e., sodium‐dependent multivitamin transporter), the resulting complex may undergo “endocytosis.” Our observations also suggest that the cellular uptake of Dox is mediated by specific interactions between the biotin transporter on cancer cells and the biotin ligand in bt‐BRNPs, underscoring the potential of this system as a specific cancer‐targeting drug‐delivery carrier.

**Figure 2:**
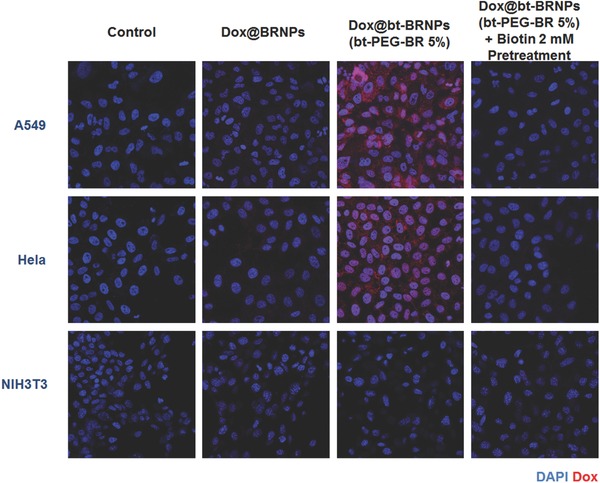
bt‐BRNPs show selectivity toward biotin‐receptor–overexpressing cell lines. Confocal microscopic images of A549 and Hela cells (biotin transporter–positive cell lines) and NIH3T3 cells (biotin transporter–negative cell line) treated with Dox@BRNPs (10 µm Dox; 10 µm PEG‐BR), Dox@bt‐BRNPs [10 µm Dox; 10 µm PEG‐BR + 5% bt‐PEG‐BR)] or medium for 2 h, with or without pretreatment with 2 × 10^−3^ m biotin.

We next examined whether Dox@bt‐BRNPs are capable of releasing drugs in response to ROS. Accordingly, Dox@bt‐BRNPs were incubated with different concentrations of 2,2′‐azobis(2‐amidinopropane) dihydrochloride (AAPH), a peroxy radical precursor, and drug release was monitored by measuring Dox fluorescence. As shown in **Figure** **3**a, almost 100% of Dox was released within 10 min after exposure to peroxy radicals, whereas no drug release was observed in the absence of radicals, indicating that bt‐BRNPs have excellent ROS‐responsive drug‐release properties. It is generally accepted that both cancer cells and the surrounding TME exhibit high oxidative stress compared with normal tissues.[[qv: 1b,c,4]] The comparison of intracellular ROS levels between HeLa, A549, and NIH3T3 cells using the ROS‐detection dye, dichlorofluorescin diacetate revealed that HeLa cells showed much higher intracellular ROS levels than NIH3T3 and even A549 cancer cells (Figure S4, Supporting Information), suggesting that the HeLa cell line is suitable for exploring the therapeutic potential of Dox@bt‐BRNPs.^14^ Consistent with the ROS levels in each cancer cell type, nuclear localization of Dox after treatment with Dox@bt‐BRNPs was faster in HeLa cells than was the case in A549 cells, suggesting the possibility of ROS‐mediated particle disruption and drug release in either an extracellular or cytosolic environment (Figure 3b).

**Figure 3:**
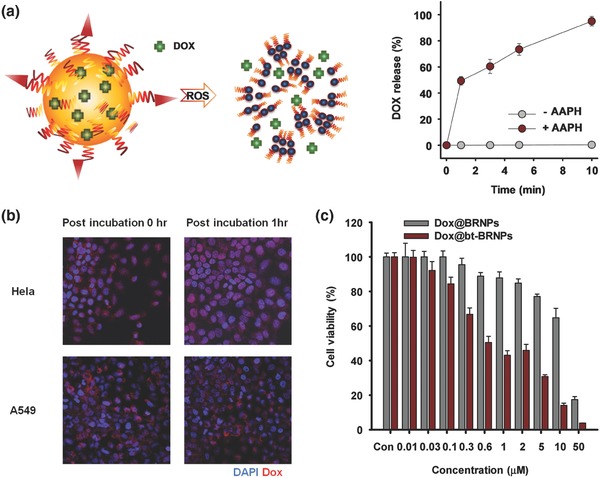
Dox‐loaded bt‐BRNPs (Dox@bt‐BRNPs) release Dox in response to ROS and exert synergistic anticancer activity in vitro. a) Dox released from Dox@bt‐BRNPs in the absence and presence of the peroxy radical generator, AAPH (100 × 10^−3^ m), at 37 °C. Data are presented as means ± standard deviations (*n* = 5). b) Confocal fluorescence images of Hela cells and A549 cells treated with Dox@bt‐BRNPs [10 µm Dox; 10 µm (95% PEG‐BR + 5% bt‐PEG‐BR)] for 1 h, with or without a 1 h post‐incubation with cell culture medium. Red, Dox fluorescence; blue, nuclei (DAPI staining). c) Viability of Hela cells incubated for 2 h with different concentrations of Dox@BRNPs and Dox@bt‐BRNPs, relative to control (culture medium), and then further incubated for 36 h. Data are presented as means ± s.e.m.

Next, the anticancer efficacy of Dox@bt‐BRNPs against HeLa cells was assessed using a cell viability assay. Bilirubin is known to possess intrinsic anticancer activity.^15^ However, our in vitro cell viability tests showed that BRNPs possessed little anticancer activity (Figure S5, Supporting Information). This is presumably attributable to the limited intracellular uptake of the highly PEGylated, hydrophilic BRNPs compared with the freely cell‐permeable hydrophobic BR. Unlike BRNPs, however, we expected that bt‐BRNPs might have anticancer activity, given their ability to undergo the transporter‐mediated internalization into cells. Consistent with these expectations, we found that, at high concentrations (>≈50 µm) bt‐BRNPs showed some degree of anticancer activity toward HeLa and A549 cells, but not toward NIH3T3 cells (Figure S5, Supporting Information). These results suggest that Dox@bt‐BRNPs exhibits synergistic anticancer activity, reflecting the combined cytotoxic effects of Dox and bt‐BRNPs. Indeed, Dox@bt‐BRNPs showed more potent cytotoxicity against HeLa cells (IC_50_ = 0.96 µm) than both free Dox (IC_50_ = 3.28 µm) and Dox@BRNPs (IC_50_ = 13.7 µm) (Figure 3c). These results indicate that the greater anticancer activity of Dox@bt‐BRNPs compared with free Dox may be a combined effect of the released Dox and the intrinsic anticancer action of bt‐BRNPs.

We next evaluated the cancer‐targeting ability of Dox@bt‐BRNPs in mice harboring biotin transporter–positive, ROS‐overproducing HeLa cell tumors. For in vivo fluorescence imaging, we incorporated the NIR dye cypate into bt‐BRNPs, yielding Cyp@bt‐BRNPs.^16^ Cyp@bt‐BRNPs or Cyp@BRNPs were intravenously injected into tumor‐bearing mice (*n* = 3/group) and whole‐body fluorescence images were taken 12 h after injection. As shown in **Figure** **4**a, much higher fluorescence intensity was evident in the tumor area of a representative mouse treated with Cyp@bt‐BRNPs compared with that in mice treated with Cyp@BRNPs. Ex vivo imaging of dissected tumors and other major organs further verified the clear difference in tumor uptake and biodistribution between the two BRNPs (Figure 4b). Cyp@bt‐BRNPs showed much higher uptake in tumors and much lower uptake in other organs, such as liver and lung, compared with Cyp@BRNPs, suggesting that the former system would exhibit superior antitumor efficacy with lower toxicity than the latter system.

**Figure 4:**
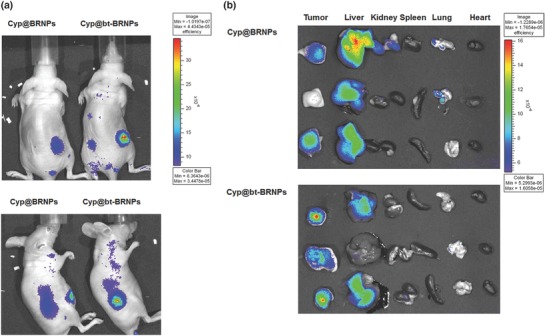
Dox@bt‐BRNPs show enhanced in vivo ability to target xenograft tumors in mice formed from biotin transporter–overexpressing Hela cells. a) In vivo fluorescence images of mice after a 4 h treatment with cypate@BRNPs (23 µg cypate; 460 µg BRNPs) or cypate@bt‐BRNPs (23 µg cypate; 470 µg bt‐BRNPs). b) Ex vivo fluorescence images of major organs (tumor, liver, kidney, spleen, lung, and heart) 14 h after treatment with cypate@BRNPs or cypate@bt‐BRNPs.

Having confirmed the enhanced anticancer activity in vitro and tumor‐targeting ability in vivo of bt‐BRNPs, we compared the therapeutic efficacy of Dox@bt‐BRNPs with that of free Dox, bt‐BRNP vehicle only, and Dox@BRNPs in HeLa tumor‐bearing mice (*n* = 5/group) after intravenous injection. Dox@bt‐BRNPs showed significantly greater antitumor efficacy, inhibiting tumor growth by ≈93% compared with that in the control PBS‐treated group; by comparison, bt‐BRNP vehicle, free Dox, and Dox@BRNPs inhibited tumor growth by ≈36%, ≈43%, and ≈65%, respectively (**Figure** **5**a,b). These values are in good agreement with the results of experiments on cancer cell binding, specific anticancer activity in vitro, and cancer targeting in vivo. It should be noted that the high antitumor potency and efficacy of Dox@bt‐BRNPs was not accompanied by body weight loss (unlike free Dox, which caused an appreciable decrease), suggesting that Dox@bt‐BRNPs are a safe delivery system (Figure 5c). Notably, bt‐BRNP vehicles alone exerted appreciable antitumor efficacy in vivo, in good agreement with their anticancer activity against HeLa cells in vitro. Furthermore, TUNEL (terminal deoxynucleotidyl transferase dUTP nick‐end labeling) assays revealed clear evidence of massive apoptosis in tumor tissue of mice treated with Dox@bt‐BRNPs compared with that of other groups (Figure 5d). These findings suggest that the potent antitumor efficacy of Dox@bt‐BRNPs is the sum of the anticancer activity of Dox and bt‐BRNPs.

**Figure 5:**
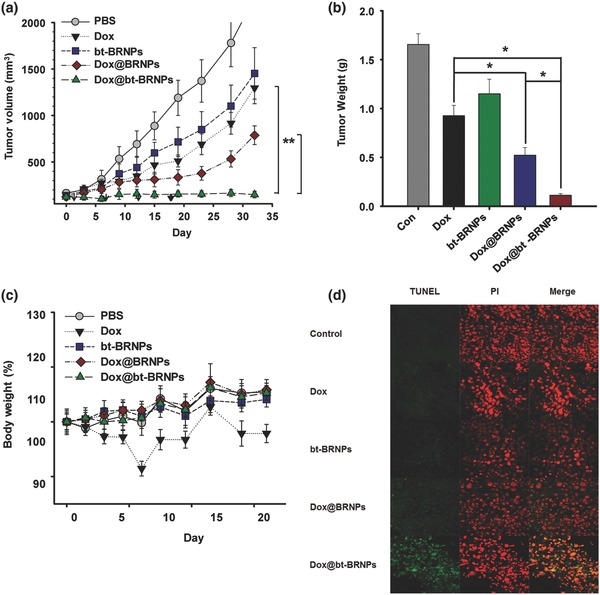
Dox@bt‐BRNPs exert improved anticancer activity in vivo. a–d) Mice bearing Hela tumors (size >100 mm^2^) were intravenously administered PBS (control), free Dox (4 mg kg^−1^), BRNPs (40 mg kg^−1^), Dox@BRNPs (40 mg kg^−1^; equivalent to 4 mg Dox/kg), or Dox@bt‐BRNPs (40 mg kg^−1^; equivalent to 4 mg Dox/kg). Tumor volume (a), body weight (b), and tumor weight (c) were measured on predetermined days. d) The degree of tumor apoptosis was compared between groups using a TUNEL assay. Data are presented as means ± s.e.m. (*n* = 5; *P < 0.05, **P < 0.001, one‐way ANOVA).

In conclusion, we reported the development of Dox@bt‐BRNPs, a cancer‐targeting, TME‐associated, ROS‐responsive DDS for effective cancer therapy. In this system, biotin was used as a ligand to target biotin transporter–overexpressing cancer cells and BRNPs were employed as a ROS‐responsive drug‐delivery carrier. Indeed, Dox@bt‐BRNPs showed a highly accelerated drug‐release profile in response to various ROS and was also highly cytotoxic toward the biotin transporter–positive HeLa cancer cell line. Once intravenously injected, Dox@bt‐BRNPs preferentially accumulated in HeLa cell tumors in mice, presumably through both passive EPR effects and specific biotin‐biotin transporter interactions, consequently resulting in greater antitumor efficacy than free Dox and Dox@BRNPs. The nanomedicine demonstrated here is a unique DDS in which the encapsulated drugs are released in response to ROS and ultimately become internalized in cancer cells, leading to high antitumor efficacy. In fact, there have been a number of reports of DDS with ROS‐responsive drug‐release characteristics. However, unlike previously reported ROS‐responsive DDS comprising de novo‐synthesized artificial materials, the present bt‐BRNPs consist of a naturally occurring biocompatible and biodegradable material, show rapid drug‐release behavior in response to various endogenous ROS, and possess intrinsic anticancer activity. Collectively, these attributes lead to potent antitumor efficacy, strengthening the clinical translational potential of this DDS. Taken together, our findings suggest that the present TME ROS‐responsive DDS will make a significant contribution to the treatment of various cancers.

## Experimental Section

*Synthesis of PEGylated Bilirubin (PEG‐BR) and Biotin‐PEGylated Bilirubin (bt‐PEG‐BR)*: PEGylated bilirubin was prepared as described previously.[[qv: 8b]] Biotin‐PEGylated bilirubin was prepared by dissolving (ZZ)‐bilirubin‐IX‐alpha (0.5 mmol) (Tokyo Chemical Industry, Tokyo, Japan) and of EDC (0.4 mmol) [1‐ethyl‐3‐(3‐dimethylaminopropyl)carbodiimide; Sigma‐Aldrich, St. Louis, MO, USA] in dimethyl sulfoxide (5 mL) (DMSO). After stirring for 10 min at room temperature, biotin‐mPEG3,400‐NH2 (0.2 mmol) (Nanocs, Boston, MA), and triethylamine (150 µL) were added to the mixture, and the reaction was allowed to proceed with stirring for 4 h at room temperature under a nitrogen atmosphere. Chloroform (450 mL) was added to the reaction mixture, which was then washed with 0.1 m HCl and brine using a separation funnel. The organic layer was collected and concentrated under vacuum. For removal of free bilirubin, 45 mL of methanol was added to the concentrated reaction mixture and the solution was centrifuged at 2000 × *g* for 10 min, after which the resulting precipitate was discarded and the supernatant was evaporated. Ether was then added to the concentrated reaction mixture, and the resulting precipitate was dissolved in chloroform for subsequent purification by column chromatography on silica using chloroform:methanol (85:15) as the mobile phase. The solvents were evaporated to yield PEG‐BR, which was subsequently subjected to ^1^H‐NMR and MALDI‐TOF (matrix‐assisted laser desorption/ionization‐time of flight) spectroscopy. ^1^H‐NMR spectra were obtained using an AVANCE400 system (Bruker Daltonics, Bremen, Germany); chemical shifts represent ppm downfield from tetramethylsilane. MALDI‐TOF spectra were obtained using an Autoflex III MALDI‐TOF system (Bruker).

*Preparation of Biotin‐Functionalized Bilirubin Nanoparticles (bt‐BRNPs)*: PEG‐BR (2 µmol) and differing proportions of bt‐PEG‐BR were dissolved in chloroform (200 µL), dried under a stream of nitrogen gas, and further dried under a vacuum to yield a film layer. For formulation of nanoparticles, the film layer was hydrated with PBS (500 µL) (137 × 10^−3^ m NaCl, 2.7 × 10^−3^ m KCl, 10 × 10^−3^ m Na_2_HPO_4_, 2 × 10^−3^ m KH_2_PO_4_), and the resulting suspension was sonicated for 10 min to yield uniform‐sized, biotin‐functionalized bilirubin nanoparticles (bt‐BRNPs). The size and zeta potential values of bt‐BRNPs were characterized using a Nanosizer ZS90 (Malvern Instruments, Ltd., Malvern, UK). BRNP morphology was monitored by transmission electron microscopy using a Tecnai TF30 ST instrument (FEI Co., Hillsboro, OR). For all subsequent in vitro and in vivo analyses, a diluted bt‐BRNP solution was used.

*Drug Encapsulation*: PBS (pH 7.4) containing Dox (LC Laboratories, Woburn, MA, USA) was added to a film layer of PEG‐BR together with various proportions of bt‐PEG‐BR, after which the solution was sonicated for 10 min. Dox‐loaded bt‐BRNPs (Dox@BRNPs) were purified by gel filtration using a Sepharose CL‐4B column (Sigma‐Aldrich) equilibrated with 20 × 10^−3^ m HEPES‐buffered 5% glucose. The amount of Dox loaded in bt‐BRNPs was quantified by first treating nanoparticles with 0.5% Triton‐X and then measuring Dox concentration by high‐performance liquid chromatography (HPLC), using a fluorescence excitation wavelength of 480 nm and a detection wavelength of 550 nm. Encapsulation efficiency and drug loading efficiency were calculated according to the following equations:

Encapsulation efficiency (%) = (weight of drug in particle/weight of drug added initially) × 100.

Drug loading percentage (%) = [weight of drug in particle/(weight of drug in particle + weight of PEG‐BR added initially)] × 100.

*Drug Release from BRNPs Induced by ROS Exposure*: The drug‐release profile was measured by exposing Dox@bt‐BRNPs (1 mg mL^−1^, 10% Dox loading percentage) to AAPH (100 × 10^−3^ m) at 37 °C. At each time point (0, 1, 3, 5, and 10 min), the amount of Dox released into the solution was monitored by measuring fluorescence using an excitation wavelength of 480 nm and an emission wavelength of 550 nm.

*Cell Culture*: The A549 human alveolar basal epithelial carcinoma cell line, Hela cervical cancer cell line, and NIH3T3 fibroblast cell line were obtained from the Korean Cell Line Bank (Seoul, Korea). NIH3T3 cells were cultured in RPMI‐1640 medium (Welgene, Daegu, Korea) containing 10% (v/v) heat‐inactivated fetal bovine serum (FBS), 100 IU mL^−1^ penicillin, and l‐glutamine in a humidified 5% CO_2_ atmosphere at 37 °C. A549 cells were cultured in Ham's F‐12K medium (Welgene) supplemented with 10% FBS in a humidified 5% CO2 atmosphere at 37 °C. Hela cell lines were cultured in Dulbecco's modified Eagle medium (DMEM; Welgene, Daegu, Korea) in a humidified 5% CO2 atmosphere at 37 °C. All cell lines were tested for mycoplasma contamination.

*Confocal Microscopy*: A549 cells, Hela cells, or NIH 3T3 cells were cultured on cover slips in 24‐well plates (1 × 10^4^ cells per well) for 24 h at 37 °C, and then incubated with culture medium (control), Dox (10 µm), Dox@BRNPs (Dox 10 µm; BRNPs 10 µm), Dox@bt‐BRNPs (Dox 10 µm; BRNPs 10 µm; 1–10% bt‐PEG‐BR) or Dox@bt‐BRNPs, with or without pretreatment with 2 × 10^−3^ m biotin at 37 °C for 1 h. Cells were fixed immediately with 4% paraformaldehyde or incubated for an additional 1 h before fixing, and then stained with 4′,6′‐diamidino‐2‐phenylindole (DAPI; Sigma‐Aldrich) and mounted with fluorescence mounting medium (Dako, Glostrup, Denmark). Cellular fluorescence was visualized using confocal laser‐scanning microscopy (LSM 710; Carl Zeiss Microimaging, Oberkochen, Germany) by exciting at 480 nm and collecting emitted fluorescence at 530–670 nm.

*MTT Assay*: A549 cells, Hela cells, or NIH 3T3 cells were cultured in 96‐well plates (0.7 × 10^3^ cells per well) at 37 °C. After incubating for 24 h and removing medium, cells were treated with fresh medium (control) or different concentrations of free Dox, BRNPs, bt‐BRNPs, Dox@BRNPs, or Dox@bt‐BRNPs for 2 or 4 h at 37 °C. The cells were then washed with PBS and incubated with fresh medium for an additional 24 or 36 h at 37 °C. After the medium was removed, cells were treated with 100 µL of fresh culture medium containing 20 µL of MTT [3‐(4,5‐dimethylthiazol‐2‐yl)‐2,5‐diphenyltetrazoliumbromide] solution (5 mg mL^−1^ in PBS) for 3 h. Thereafter, 200 µL of DMSO was added to each well to dissolve the resulting formazan crystals and each well was mixed by pipetting to ensure complete formazan crystal dissolution. Finally, absorbance was measured at 540 nm using a 96‐well plate reader.

*Measurement of Intracellular ROS*: NIH3T3, A549, and Hela cell lines were plated in 96‐well plates at a density of 3 × 10^4^ per well in the corresponding standard culture medium. After 4 h, the medium was replaced and 30 µm of DCF‐DA, an oxidant‐sensitive fluorescent dye, was added to each well. After incubating for 1 h and washing with PBS, adherent cells were lysed in 1 mL^−1^ of RIPA buffer and analyzed immediately using a Perkin Elmer Fluorescence Spectrophotometer 650‐10S equipped with a Xenon Power Supply (excitation 488 nm, emission 510 nm). Data were normalized to total protein content.

*Animals*: All animals were obtained from Orient Bio, Inc. (Seongnam, Korea) and were housed under pathogen‐free conditions in the animal facility at the Korea Advanced Institute of Science and Technology. Mice were assigned randomly to experimental groups. The experiments themselves were not randomized, and investigators were not blinded to allocation during experiments or outcome assessments unless each section specifically included a blind assessment. All surgeries were performed under isoflurane anesthesia, and all effort was made to minimize suffering. All animal procedures were reviewed and approved (approval number: KA2013‐24) by the Korea Advanced Institute of Science and Technology Institutional Animal Care and Use Committee (KAIST‐IACUC) for compliance with ethical procedures and standards of scientific care.

*Analysis of the Targeting of bt‐BRNPs to Tumor Sites*: A tumor xenograft mouse model was prepared by injecting 1 × 10^6^ Hela cells subcutaneously into the dorsal flanks of 6‐week‐old female BALB/c nude mice. When tumor volumes reached at least 1000 mm^3^, cypate@BRNPs (23 µg cypate; 460 µg BRNPs) or cypate@bt‐BRNPs (23 µg cypate; 470 µg bt‐BRNPs) was intravenously injected via the tail vein. Cypate@BRNPs and cypate@bt‐BRNPs were generated by preparing film layers containing cypate and PEG‐BR, or cypate, PEG‐BR and bt‐BRNPs, and subsequently rehydrating them with PBS to yield cypate@BRNPs and cypate@bt‐BRNPs, which were purified by Sephadex 4G column chromatography. The loaded amount of cypate was measured by HPLC analysis. At a predetermined time, in vivo fluorescence images of mice were acquired under isoflurane anesthesia using a Xenogen Lumina In Vivo Imaging System (IVIS; PerkinElmer, Waltham, MA, USA) with an ICG filter channel and an exposure time of 5 s. After 24 h, mice were sacrificed, and the major organs (colon, kidney, liver, spleen, lung, and heart) were collected. The fluorescence intensities of organs from each group were analyzed using the Xenogen Lumina IVIS (PerkinElmer, Waltham, MA, USA) with an ICG filter channel and an exposure time of 5 s.

*Anticancer Activity*: Anticancer efficacy in vivo was investigated after tumor volumes had reached at least 100 mm^3^ (day 0), at which point tumor‐bearing mice were randomly divided into four groups (*n* = 5 per group), while minimizing weight and tumor size differences, and then administered 100 µL of Dox (4 mg kg^−1^), BRNPs (40 mg kg^−1^), Dox@bt‐BRNPs (4 mg kg^−1^ Dox; 40 mg kg^−1^ BRNPs; 10% mole percentage of bt‐PEG‐BR), or Dox@BRNPs (4 mg kg^−1^ Dox; 40 mg kg^−1^ BRNPs) by intravenous injection five times on predetermined days (day 0, 3, 6, 9, and 12); mice injected with PBS served as a control. Tumor dimensions in each group were measured on predetermined days using a Vernier caliper. Tumor volume was calculated according to the following formula: volume = (length × width × height)/2. The percentage of tumor growth inhibition on the final day was calculated as [(TvolControl – TvolTreatment)/TvolControl] × 100, where Tvol is the final tumor volume minus the initial tumor volume. Body weights were also monitored on predetermined days. Mice were sacrificed 32 d after the first treatment and tumors were collected. Tumor weights in each group were measured, and apoptotic cells in tumor sections were detected by TUNEL assay (Bio Vision, Milpitas, CA, USA) according to the manufacturer's protocol.

*Statistical Analysis*: All experiments were performed twice independently. The results are expressed as means ± standard errors of the mean (s.e.m.) or standard deviations (s.d.). One‐way ANOVA followed by Dunnett's post hoc test was used for evaluating differences among groups. *P*‐values <0.05 were considered significant. XLSTAT Software (Addinsoft, Inc., New York, NY, USA) was used for all statistical analyses.

## Conflict of Interest

The authors declare no conflict of interest.

## Supporting information

Supplementary
