# Supplementary material for: Biotinylated Bilirubin Nanoparticles as a Tumor Microenvironment‐Responsive Drug Delivery System for Targeted Cancer Therapy
Source: Adv Sci (Weinh). 2018 Apr 24;5(6):1800017. doi: 10.1002/advs.201800017 (PMC6010876; doi:10.1002/advs.201800017)
Supplement: Supplementary file 1 — Supplementary [file ADVS-5-1800017-s001.pdf]

Copyright WILEY-VCH Verlag GmbH & Co. KGaA, 69469 Weinheim, Germany, 2018.

ADVANCED SCIENCE
Open Access

Supporting Information

for *Adv. Sci.,* DOI: 10.1002/advs.201800017

Biotinylated Bilirubin Nanoparticles as a Tumor Microenvironment-Responsive Drug Delivery System for Targeted Cancer Therapy

*Yonghyun Lee, Soyoung Lee, and Sangyong Jon**

Copyright WILEY-VCH Verlag GmbH & Co. KGaA, 69469 Weinheim, Germany, 2016.

Supporting Information

Biotinylated Bilirubin Nanoparticles as a Tumor Microenvironment-responsive Drug Delivery System for Targeted Cancer Therapy

*Yonghyun Lee, Soyoung Lee, and Sangyong Jon**

KAIST Institute for the BioCentury, Department of Biological Sciences, Korea Advanced Institute of Science and Technology, 291 Daehak-ro, Daejeon 305-301, Republic of Korea.
**e-mail: syjon@kaist.ac.kr.*

Contents

1. Figure S1. Characterization of biotin-PEG-bilirubin (bt-PEG-BR) nanoparticles.
2. Figure S2. Scheme for the formulation of biotin-conjugated bilirubin nanoparticles (bt-BRNPs) with or without Dox, and determination of hydrodynamic size and zeta potential values of bt-BRNPs.
3. Figure S3. Bt-BRNPs formulated with 95% PEG-BR and 5% bt-PEG-BR show the best ability to target biotin-receptor–overexpressing cell lines.
4. Figure S4. Bt-BRNPs show specific cytotoxicity towards biotin-receptor–positive cell lines.
5. Figure S5. Hela cells have the highest levels of ROS among A549, Hela and NIH3T3 cell lines.

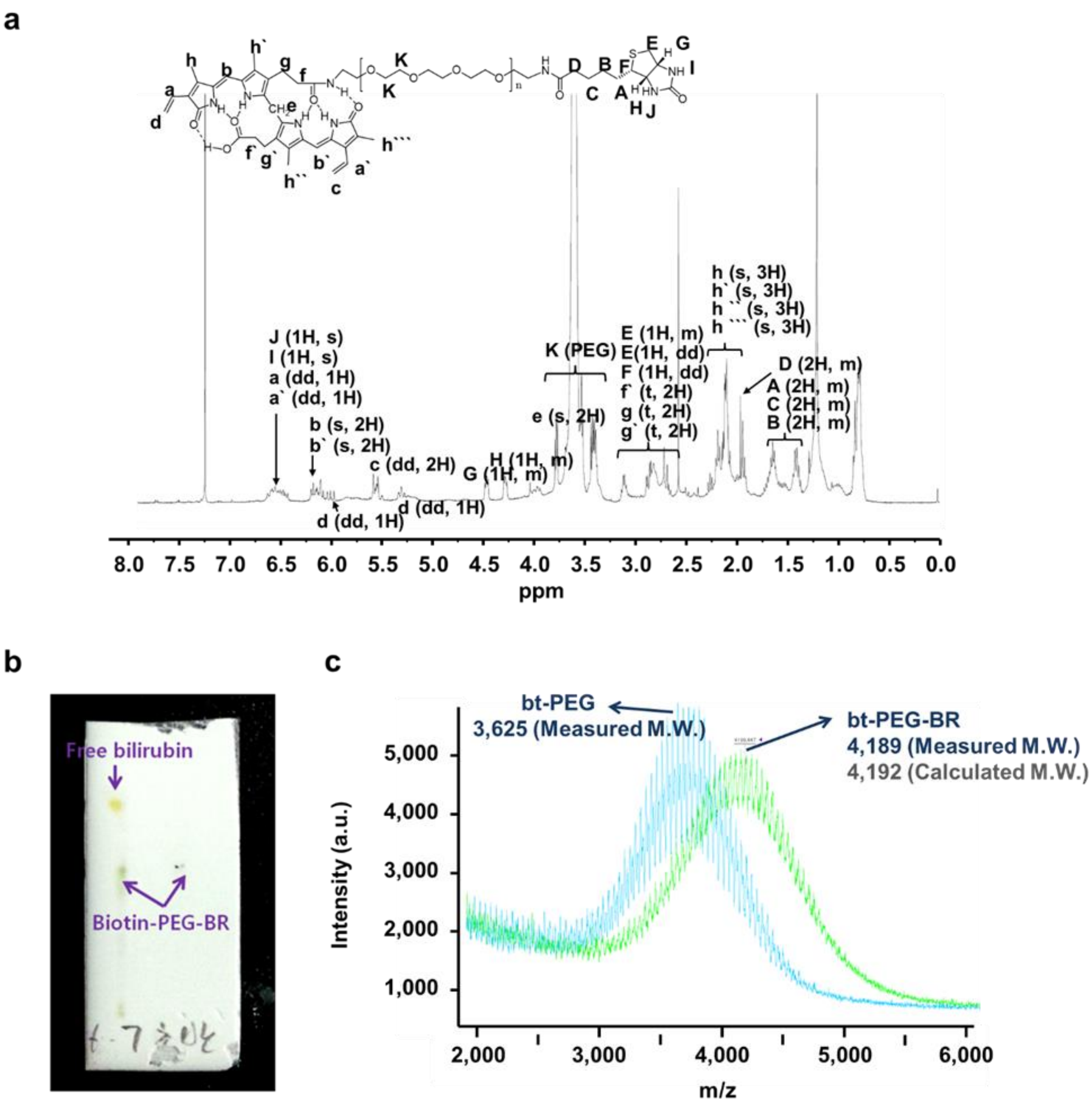

Supplementary Figure 1. **Characterization of biotin-PEG-bilirubin (bt-PEG-BR). a**, ^{1}H-NMR spectrum of bt-PEG-BR in DMSO-d6. **b–c**, TLC peak (**b**) and MALDI-TOF/MS spectrum (**c**) of PEG-BR.

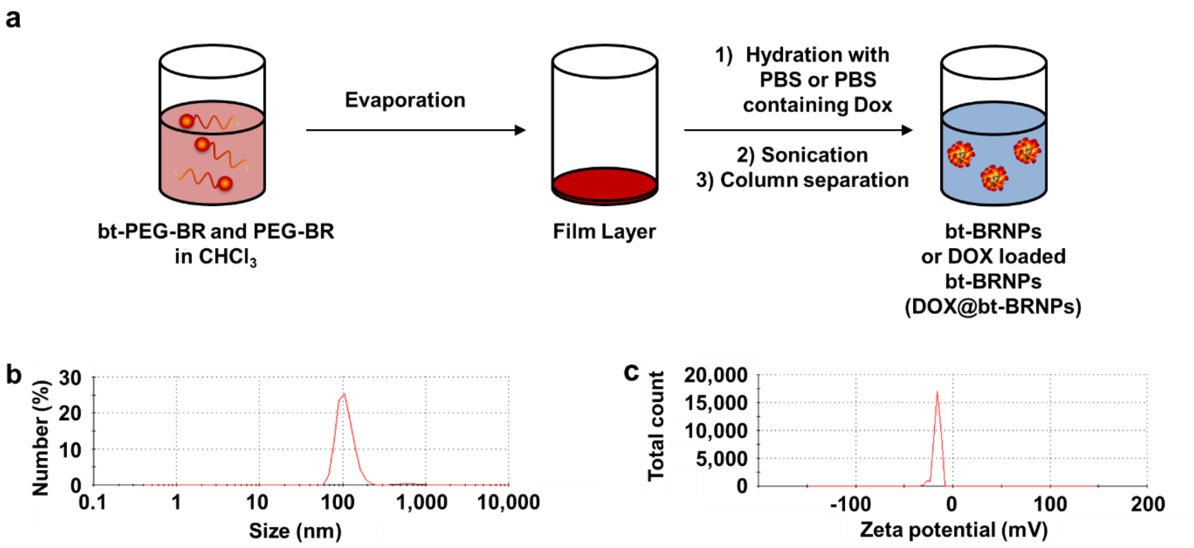

Supplementary Figure 2. **a**, Scheme for the formulation of biotin-conjugated bilirubin nanoparticles (bt-BRNPs), with or without Dox. **b and c**, Hydrodynamic size (**b**) and zeta potential value (**c**) of bt-BRNPs.

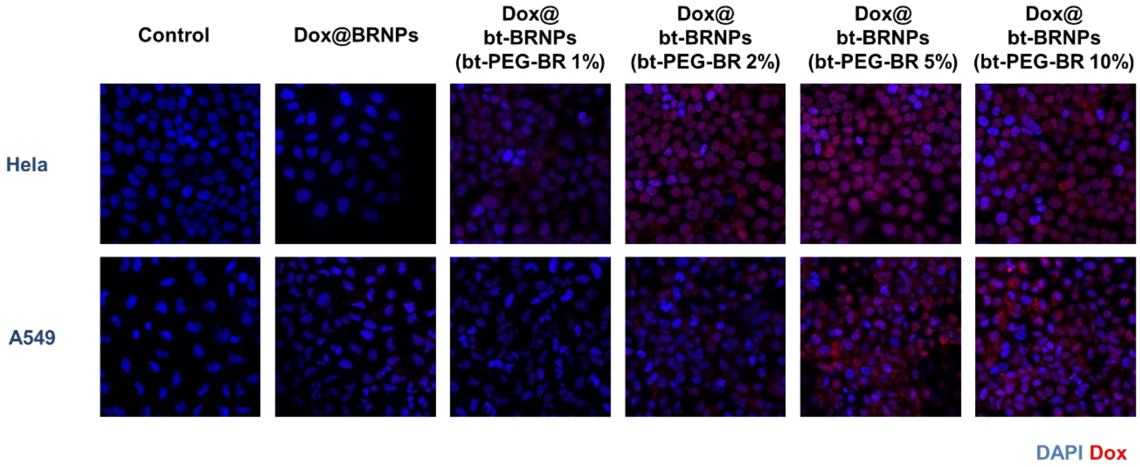

Supplementary Figure 3. **Bt-BRNPs formulated from 95% PEG-BR and 5% bt-PEG-BR show the best ability to target biotin-receptor–overexpressing cell lines**. Confocal microscopic images of A549 and Hela cells (biotin receptor-positive–cell lines) treated for 2 h with Dox@BRNPs, Dox@bt-BRNPs formulated with various percentages of bt-BRNPs or medium.

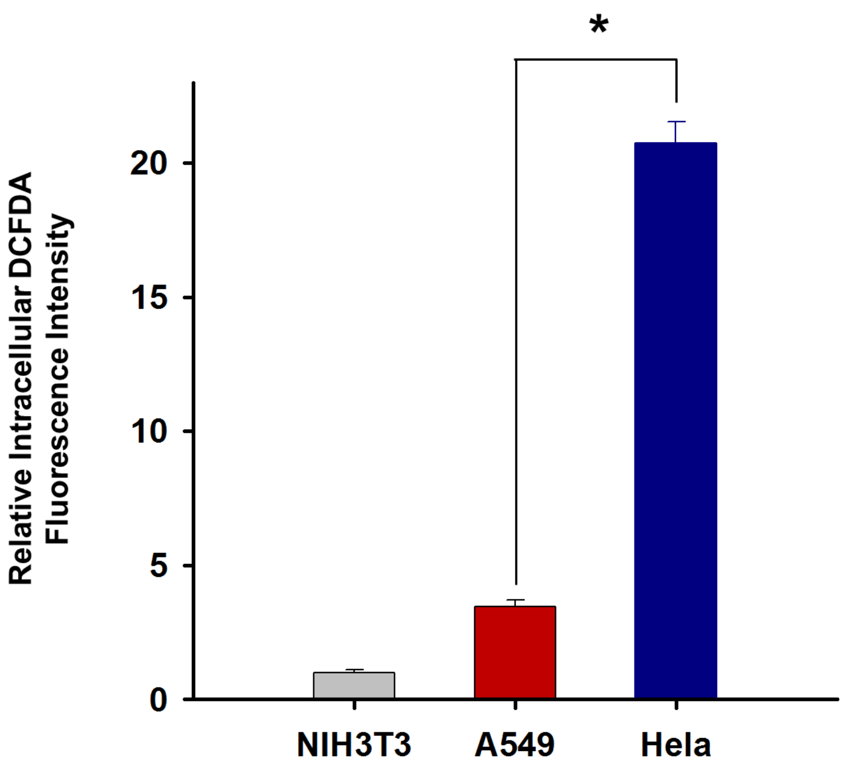

Supplementary Figure 4. Hela cells have the highest levels of ROS among A549, Hela, and NIH3T3 cell lines. Comparison of the correlation of ROS levels (determined using DCFDA dye) with fluorescence intensity in A549, Hela, and NIH3T3 cells. Data are presented as means ± s.e.m. (n = 5; *P < 0.05, one-way ANOVA).

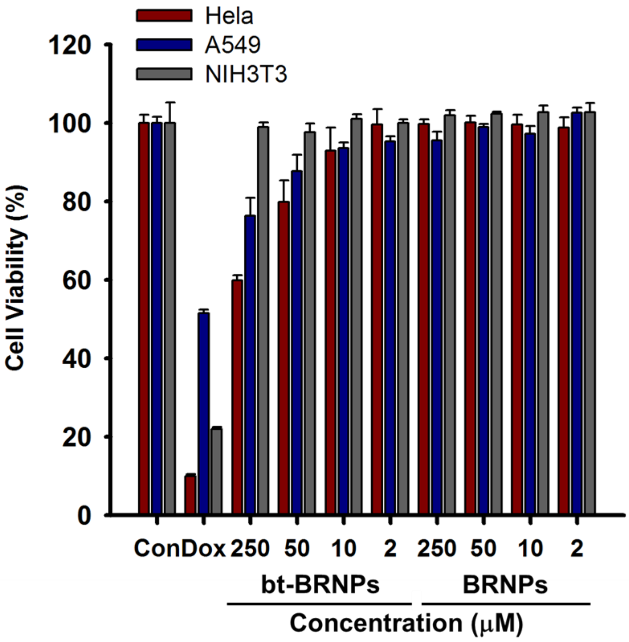

Supplementary Figure 5. **Bt-BRNPs exert cytotoxicity specifically against biotin-receptor–positive cell lines.** Viability of Hela, A549, and NIH3T3 cells after a 4-h incubation with different concentrations of bt-BRNPs and BRNPs and an additional 16-h incubation with culture medium. Free 10 μM Dox was used as a positive control.
